# *Coulmannia rossensis* sp. n. (Isopoda, Asellota, Paramunnidae) from the Ross Sea, Southern Ocean

**DOI:** 10.3897/zookeys.82.775

**Published:** 2011-02-23

**Authors:** Madhumita Choudhury, Brenda L. Doti, Angelika Brandt

**Affiliations:** 1 Zoological Institute and Museum, University of Hamburg, Martin-Luther-King-Platz 3, 20146 Hamburg, Germany; 2 Departamento de Biodiversidad y Biología Experimental, Facultad de Ciencias Exactas y Naturales, Universidad de Buenos Aires, Ciudad Universitaria, C1428EHA, Buenos Aires, Argentina

**Keywords:** Isopoda, *Coulmannia*, new species, taxonomy, Ross Sea, Southern Ocean

## Abstract

A new species of Coulmannia, Coulmannia rossensis, is described from the Ross Sea, Antarctica. It is most similar to Coulmannia ramosae Castelló, 2004, but can easily be distinguished from this species bythe males yielding a pair of granulate humps on the dorsum of the pereonites 1–6 and a single granulate hump on the pereonite 7 and the free pleonite. Coulmannia rossensis **sp. n.** is sexually dimorphic. The dorsal sculpture of the female bodies yield a single granulate hump on all the pereonites and free pleonite. The species of the genus Coulmannia are restricted to the Southern Ocean, and Coulmannia rossensis **sp. n.** is the fourth species included in it.

## Introduction

Paramunnidae Vanhöffen, 1914 is a large family of asellote isopods; at present it includes 42 genera, many of them recently erected (see Just and Wilson 2004, 2006, 2007; Doti et al. 2009; Just 2009a, b; Shimomura 2009). This family has a worldwide distribution, but with an overwhelming preponderance of species in temperate to cold water of the Southern Hemisphere (Wilson 1980; Just and Wilson 2004). In agreement with this observation, Choudhury and Brandt (2007) found Paramunnidae to be one of the most abundant and frequent families among the Isopoda collected in the Ross Sea with RV *Italica* in 2004. A preliminary study of this collection showed that many of the species found in this survey were new to science (Choudhury and Brandt 2009). Based on this material, a new paramunnid of the genus Coulmannia is described.

To date species in the genus Coulmannia have been exclusively reported from the Southern Ocean. Hodgson (1910) erected the genus Coulmannia to include two new species from the Ross Sea, viz.: Coulmannia australis Hodgson, 1910 from Coulman Island and Coulmannia frigida Hodgson, 1910 from McMurdo Sound. More recently, Castelló (2004a) described the third species of the genus, Coulmannia ramosae Castelló, 2004 from the South Shetland Islands. In the present paper Coulmannia rossensis sp. n. is described from the Ross Sea. The morphological differences of the four species belonging to this genus are discussed and presented in a table, an identification key is offered.

## Material and methods

Specimens of Coulmannia rossensis sp. n. were collected during the 19^th^ Antarctic expedition to the Ross Sea on board the RV *Italica*, in February 2004. Samples were taken along a latitudinal transect between Cape Adare and Terra Nova Bay with a modified Rauschert dredge (Lörz et al. 1999). The material was sieved using a 500 µm mesh and fixed in pre-cooled 96% ethanol for later DNA analysis.

For the taxonomic description some specimens were stained with Chlorazole Black E®, and their appendages were dissected and temporarily mounted in glycerine. Pencil drawings of the whole animal and dissected appendages were prepared using a Carl Zeiss (Axioskop 2) compound microscope equipped with a camera lucida. Digital illustrations were made with a Wacom tablet and the Adobe Illustrator program following Coleman (2003).

The length of the head, pereonites, free pleonite, and pleotelson were all estimated along the mid-dorsal line. The width of the head was measured between the tips of the eyestalks. Body length as well as lengths of the articles of the appendages were measured according to Hessler (1970).

The material examined of Coulmannia rossensis sp. n. is lodged at the Zoological Museum of Hamburg (ZMH). For comparison purposes, the type material of Coulmannia ramosae Castelló, 2004 (holotype male MZB 2003-1229A and paratype male MZB 2003-1229B) deposited in the Museum of Zoology, Barcelona (MZB) was also examined.

## Taxonomy

### Family Paramunnidae Vanhöffen, 1914

#### Genus Coulmannia

Hodgson, 1910

##### Composition.

Coulmannia australis Hodgson, 1910; Coulmannia frigida Hodgson, 1910; Coulmannia ramosae Castelló, 2004 and Coulmannia rossensis sp. n.

##### Key to species of Coulmannia

**Table:** 

1	Lateral margins of all pereonites produced into 2 processes	Coulmannia australis Hodgson, 1910
–	Lateral margins of at least one pereonite produced into a single process	2
2	Lateral margins of pereonite 1 produced into a single process, remaining pereonites produced into 2 processes	Coulmannia frigida Hodgson, 1910
–	Lateral margin of pereonites 2–4 produced into a single process, remaining pereonites rounded	3
3	Male: pereonites 1–6 with a pair of granulate humps mid-dorsally, pereonite 7 and free pleonite with a single granulate hump. Female: all pereonites and free pleonite with a single granulate hump	Coulmannia rossensis sp. n.
–	Male: pereonites 1 and 2 with a pair of granulate humps mid-dorsally, remaining pereonites and free pleonite with a single granulate hump. Female: unknown	Coulmannia ramosae Castello, 2004

##### Coulmannia rossensis sp. n.

urn:lsid:zoobank.org:act:4F4A530A-2215-4B75-8CCB-6CF828F56EE9

Figs 1 4 

###### Material examined.

Ross Sea, RV *Italica*.

####### Holotype:

Adult male 1.7 mm (ZMH 42000-718); station H out 2, 72°17.5'S, 170°29.4'E, 353 m depth, 11 Feb 2004.

####### Paratypes,

same locality as holotype: 5 males (1.3–1.4 mm), 4 brooding females (1.6–1.7 mm), 2 females (1.2, 1.3 mm) and 2 juveniles (0.9, 1 mm); (ZMH 42000-719).

**Figure 1.**
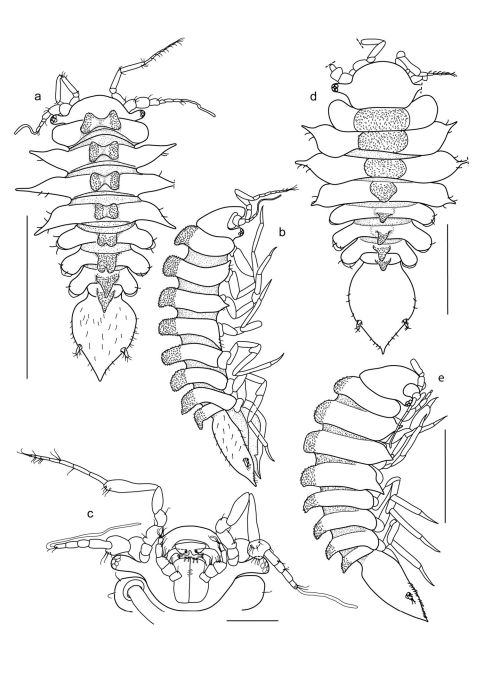
Coulmannia rossensis sp. n. Holotype male (ZMH–42000-718), **a** dorsal view **b** lateral view, **c** head in ventral view. Paratype female (ZMH–42000-719-a) **d** dorsal view. Paratype female (ZMH–42000-719-b) **e** lateral view. *Scale bars* 1 mm (a), 0.2 mm (c), 0.5 mm (d, e); a and b share the same scale.

####### Additional material:

Station H in 3, 72°17.0'S, 170°13.1'E, 316 m depth, 16 Feb 2004: 5 males, 2 brooding females, 1 female and 2 juveniles. Station H in 4, 72°17.1'S, 170°14.0'E, 196 m depth, 16 Feb 2004: 7 males, 3 females and 2 juveniles. Station H in 2, 72°16.9'S, 170°12.2'E, 391 m depth, 10 Feb 2004: 4 males and 1 female. Station SMN, 74°43.2'S, 164°13.1'E, 366 m depth, 20 Feb 2004: 2 males and 2 juveniles. Station H out 1, 72°15.7'S, 170°24.8'E, 458 m depth, 9 Feb 2004: 2 males, 1 female and 2 juveniles. Station H out 4, 72°18.5'S, 170°26.8'E, 235 m depth, 12 Feb 2004: 4 males and 2 females. Station A 4, 71°18.4'S, 170°28.9'E, 230 m depth, 14 Feb 2004: 1 female. Station C 1, 73°24.5'S, 170°23.2'E, 474 m depth, 18 Feb 2004: 1 brooding female. Station C 2, 73°22.7'S, 170°06.9'E, 410 m depth, 18 Feb 2004: 2 males, 3 brooding females, 6 females and 3 juveniles.

###### Diagnosis.

Males with dorsal sculpture formed by a pair of granulate humps on pereonites 1–6, and a single granulate hump on pereonite 7 and free pleonite. Females with single granulate hump on all pereonites and free pleonite; hump on pereonite 1 widest and shallowest, following humps gradually becoming narrower and higher towards distal end. Lateral margins of pereonites 1 and 5–7 rounded, those of pereonites 2–4 produced into single process. Coxae rounded, visible dorsally on pereonites 5–7 only.

###### Description of adult male

(body description based on the holotype male, description of appendages based on a paratype male).

####### Body

(Fig. 1a, b) total length 1.7 mm; width 0.6 length, widest at pereonite 3. Cephalon width 1.9 length (Fig. 1c), anterior margin broadly rounded. Eyestalks prominent, width 0.6 length, with 5 ommatidia. Pereonites 1–6 with two granulate humps mid-dorsally, pereonite 7 and free pleonite with a single granulate hump each. Lateral margins of pereonites 1 and 5–7 rounded, those of pereonites 2–4 produced into a single process. Coxae rounded, visible in dorsal view on pereonites 5–7 only. Pleotelson and free pleonite together 1.7 as long as last 3 pereonites combined. Pleotelson oval, lateral margins smooth and convex, apex pointed. Uropods inserted at about 2/3 of pleotelson length in posterolateral indentations.

####### Antennula

**Figure 2.**
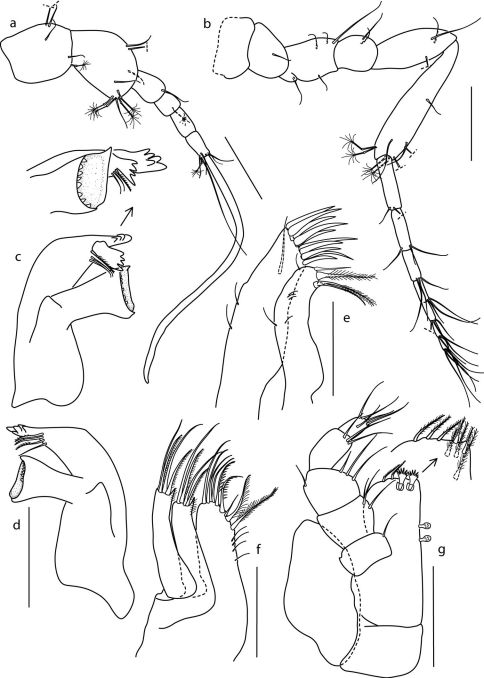
Coulmannia rossensis sp. n. Paratype male (ZMH–42000-719-c), **a** antennula **b** antenna **c** left mandible with detail of the incisor and molar processes **d** right mandible **e** maxillula **f** maxilla **g** maxilliped with detail of endite distal end (fan setae were omitted in the detail). *Scale bars* 0.1 mm (a–d, g), 0.05 mm (e, f); c and d share the same scale.

(Fig. 2a), articles 1–3 longer than wide; first article not extending beyond apex of eyestalk, with 1 penicillate and 4 simple setae; second article largest, with 4 penicillate and 5 simple setae; article 3 shorter than article 2, with 1 simple seta; article 4 shortest, with 1 penicillate seta, article 5 slightly longer than article 6, without setation; article 6 with 5 simple setae, 1 penicillate seta and 1 aesthetasc.

####### Antenna

(Fig. 2b), article 1 broken off during dissection, without setation (see Fig. 1c); article 2 with 1 simple seta; article 3 shorter than article 5, with 5 simple setae; article 4 shortest, with 2 simple setae; article 5 with 3 simple setae; article 6 longest, with 5 penicillate and 5 simple setae; flagellum with 7 articles, each article with numerous simple setae.

####### Left mandible

(Fig. 2c) stout, without palp; incisor process with 5 blunt cusps; spine row with 1 serrate and 2 simple setae; lacina mobilis 4-cusped; molar process cylindrical, transversely truncated, with lower margin of apex toothed. Right mandible (Fig. 2d) as left one, except for: spine row with 3 serrate and 1 simple setae; lacinia mobilis absent.

####### Maxillula

(Fig. 2e), lateral lobe with 10 stout cuspidate setae distally and 1 simple seta near distal margin; medial lobe with 2 simple and 2 setulate setae distally.

####### Maxilla

(Fig. 2f), lateral and medial lobes with 2 simple and 2 pectinate setae, distomedial margin with single acute projection finely setose; inner lobe with 5 simple, 2 pectinate and 2 setulate setae on distomedial margin, 4 simple slender setae on medial margin.

####### Maxilliped

(Fig. 2g), endite reaching half-length of palp article 3, with 2 coupling hooks, distal margin with 4 setulate setae (see detail), ventral surface with 1 setulate and 2 fan setae, dorsal with 3 setulate setae; epipod ovate, width 0.5 length, reaching dorsal margin of palp article 2. Palp, article 1 with 1 tooth on lateral margin, relative lengths of articles 1.0:1.4:1.4:1.7:1.0.

####### Pereopod I

**Figure 3.**
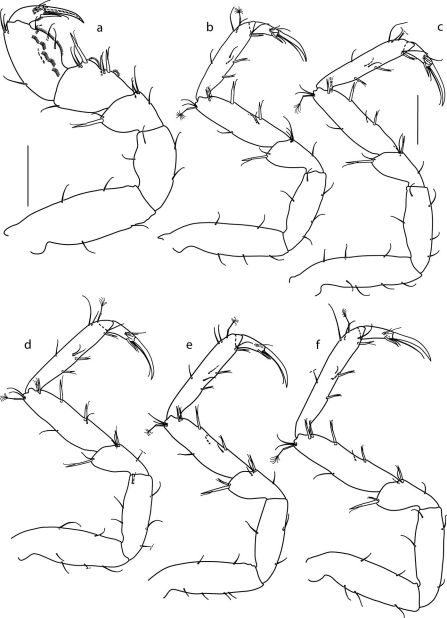
Coulmannia rossensis sp. n. Paratype male (ZMH–42000-719-c), **a** pereopod I **b** pereopod II **c** pereopod III **d** pereopod IV **e** pereopod V **f** pereopod VI. *Scale bars* 0.1 mm; b–f share the same scale.

(Fig. 3a) stoutest. Basis longest article, with 4 simple setae. Ischium with 4 simple setae. Merus with 1 robust and 1 simple setae distodorsally, ventral margin with 4 simple setae. Carpus triangular, length 0.88 ischium length, ventral margin with 2 robust and 4 simple setae and 2 cuticular combs, distodorsal margin with 1 simple seta. Propodus oval, ventral and dorsal margins with 4 simple setae each, anterior surface with 1 simple seta and cuticular combs. Dactylus with 4 simple setae near distal margin and 2 simple setae between unguis and ventral claw, unguis slightly longer than dactylus, ventral claw shorter than unguis, approximately 0.5 unguis length.

####### Pereopods II–VII

**Figure 4.**
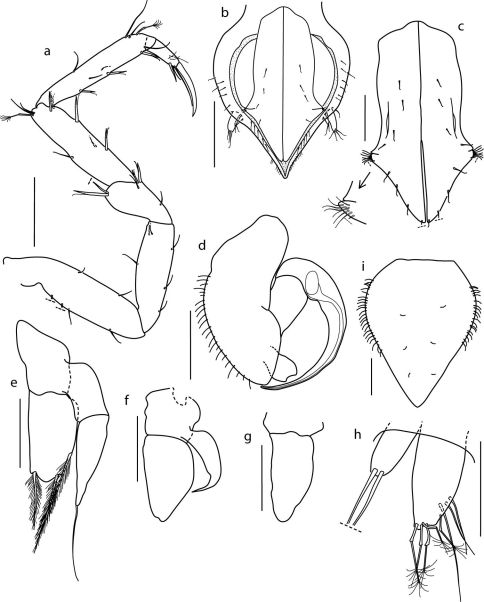
Coulmannia rossensis sp. n. Paratype male (ZMH–42000-719-c), **a** pereopod VII **b** pleotelson in ventral view **c** pleopod I **d** pleopod II **e** pleopod III **f** pleopod IV **g** pleopod V **h** uropod. Paratype female (ZMH–42000-719-a) **i** operculum. *Scale bars* 0.1 mm (a, c–g, i), 0.2 mm (b), 0.05 mm (h).

(Figs 3b–f, 4a) subequal in shape and length. Meri distodorsally with 1 robust seta on pereopods II–IV and 2 robust setae on pereopods V–VII; distoventrally with 1 robust seta on pereopods III–VI and 2 robust setae on pereopod VII. Carpi and propodi with 1 distodorsal penicillate seta each; ventral margin with 4 robust setae and 3 robust setae, respectively. Ungues 1.6–1.9 dactyli length, ventral claws 0.35 ungues length.

####### Pleopod I

(Fig. 4b, c), lateral lobes at level of 2/3 of its length, each lobe with 8 simple setae; ventral surface with 8 simple setae; distal margin with 8 simple setae.

####### Pleopod II

(Fig. 4d), sympod lateral margin rounded and setose; endopod stylet-like, curved to the apex of the sympod, relative lengths endopod: sympod, 1.0:1.3; exopod distally concave, without setae.

####### Pleopod III

(Fig. 4e), endopod width 0.6 length, with 3 plumose setae distally; exopod with 2 articles, distal one with 1 simple seta apically, extending beyond tips of endopod setae.

####### Pleopod IV

(Fig. 4f), endopod width 0.54 length, exopod reaching half length of endopod.

####### Pleopod V

(Fig. 4g) width 0.5 length.

####### Uropod

(Fig. 4h) biramous; exopod 0.4 endopod length, distally with 2 simple setae (broken in the specimen drawn); endopod with 5 penicillate and 3 simple setae.

###### Description of adult female

(Figs 1d, e; 4i). As male in body shape, except for: dorsal sculpture with a single granulate hump on all pereonites and free pleonite; hump on pereonite 1 widest and shallowest, following humps gradually becoming narrower and higher towards distal end. Operculum width 0.74 length, margins finely setose, ventral surface with 6 simple setae. Remaining appendages as those of the male.

###### Distribution.

**Figure 5.**
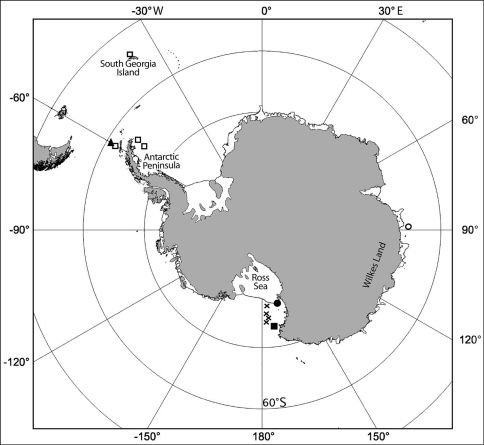
Geographic distribution of the Coulmannia species. Coulmannia rossensis sp. n. (cross); Coulmannia ramosae Castelló, 2004 (triangle); Coulmannia australis Hodgson, 1910 (square); Coulmannia frigida Hodgson, 1910 (circle). Full square and full circle stand for the type locality of the latter two species.

Only known from type locality (Fig. 5).

###### Etymology.

The species name refers to the type locality, the Ross Sea.

###### Remarks.

Because of the dorsal ornamentation and the lateral margins of the pereonites Coulmannia rossensis sp. n. is most similar to Coulmannia ramosae Castelló, 2004. The main differences between these two species are (characters found in Coulmannia ramosae in parentheses): two granulate humps on pereonites 1–6, single granulate hump on pereonite 7 and free pleonite (two granulate humps on pereonites 1 and 2, single granulate humps on remaining segments); pleotelson width 0.76 length (width 0.51 length, apex of pleotelson more produced); basis of pereopods with simple setae only (with simple and robust setae); propodi of pereopods II–IV with 3 robust setae (with 4 robust setae).

**Table 1.** Character comparison of the species included in Coulmannia Hodgson, 1910. *Abbreviations*: **Prns.** = pereonites; **PI**= first pereopod; **RS** = robust setae. * This range includes the data from the specimens described by Hodgson (1910) and Nordenstam (1933).

	*Lateral margins of pereonites*	*Dorsal sculpture of pereonites and free pleonite*		*Coxae in dorsal view*	*PI, carpus ventral margin with*	*Body length (mm)*
Coulmannia australis Hodgson, 1910	Prns. 1–7 produced into two processes	One smooth conical hump on each segment (without sexual dimorphism; José Castelló, pers. comm.)		Not visible	2–5 RS*	5–9*
Coulmannia frigida Hodgson, 1910	Prn. 1 produced into a single process, prns. 2–7 produced into two processes	One smooth conical hump on each segment(sexual dimorphism unknown)		Not visible	Unknown	3.5
Coulmannia ramosae Castelló, 2004	Prns. 1 and 5–7 rounded, prns. 2–4 produced into a single process	Two granulate humps on prns. 1–2, single granulate hump on remaining segments(sexual dimorphism unknown)		Visible on prns. 5–7 only	2 RS	2.1
Coulmannia rossensis sp. n.	Prns. 1 and 5–7 rounded, prns. 2–4 produced into a single process	♂♂: two granulate humps on prns. 1–6, single granulate hump on remaining segments	♀♀: single granulate hump on all segments, hump on prn. 1 widest and shallowest	Visible on prns. 5–7 only	2 RS	0.9–1.7

## Discussion

At present, the genus Coulmannia Hodgson, 1910 contains four species, each one with a particular arrangement in the dorsal sculpture of the body and in the lateral margins of the pereonites (see Table 1). It is worth noticing that other genera of Paramunnidae, such as Heterosignum Gamô, 1976; Meridiosignum Just and Wilson, 2007; Holodentata Doti, Choudhury and Brandt, 2009; and Pentaceration Just, 2009 also include species with different dorsal sculptures and/or lateral margins arrays. The pereonites of Coulmannia rossensis sp. n. and Coulmannia ramosae Castelló, 2004 show lateral margins similar to those present in the species of the genus Heterosignum. This genus, however, differs from Coulmannia in having long and slender eyestalks, antenna with an elongate third article, and pleotelson with denticulate margins, anteriorly narrow and cylindrical.

Coulmannia rossensis sp. n. has a remarkable sexual dimorphism in the arrangement of the dorsal sculpture. Contrary, no sexual dimorphism was found in Coulmannia australis Hodgson, 1910 (José Castelló, pers. comm.). Regarding Coulmannia frigida Hodgson, 1910, both the original description and that presented by Vanhöffen (1914) were based on a single specimen, and none of these authors mentioned the sex of the specimens examined. Thus, sexual dimorphism in Coulmannia frigida remains unknown. Similarly, Castelló (2004a) had only two males when he described Coulmannia ramose; therefore, the sexual dimorphism in this species is also unknown.

The four species belonging to Coulmannia were found exclusively in the Southern Ocean, Coulmannia australis being the most widely distributed (Fig. 5). This species was originally described by Hodgson (1910) from a single specimen collected on Coulman Island, Ross Sea at 183–400 m depth. Afterward, Nordenstam (1933) reported Coulmannia australis from the Antarctic Peninsula at 360–400 m depth, and the South Georgia Island at 252–310 m depth, and more recently Castelló (2004b) recorded it from the South Shetland Islands at 89–220 m depth. Limited distributions have been reported for many asellote species (see Hessler 1970; Just and Wilson 2004). Besides lacking free-living larvae, paramunnid species display a reduced mobility; hence, limited distributions are expected. Coulmannia australis, however, seems to have a circumpolar distribution. There are some minor differences between the specimens of Coulmannia australis described by Hodgson (1910) and Nordenstam (1933), mainly in the body length and in the number of robust setae on the ventral margin of carpus of first pereopod. The variation in the number of setae most probably is related to the body length: the specimen described by Hodgson (1910) is 5 mm long and has 2 or 3 robust setae on the carpus of pereopod I, whereas the specimen described by Nordenstam (1933) is 9 mm long and has 5 robust setae. A carefully examination of the type specimen of Coulmannia australis and those reported from other areas is required to corroborate this wide distribution.

Records from the remaining three species of Coulmannia are scarce: Coulmannia frigida was described by Hodgson (1910) from McMurdo Sound at 229 m depth, and later on reported from the Gauss Station, Wilkes Land at 385 m depth by Vanhöffen (1914); Coulmannia ramosae was found only in the South Shetland Island at 89–220 m depth by Castelló (2004a); and Coulmannia rossensis is herein reported from the Ross Sea at 196–474 m depth (Fig. 5).

## Supplementary Material

XML Treatment for Coulmannia
